# Integrative approach to profile resilience and risk factors in Alzheimer’s disease

**DOI:** 10.1002/alz.091859

**Published:** 2025-01-03

**Authors:** Ozkan Is, Jeremiah Bergman, Xue Wang, Frederick Q Tutor‐New, Ozkan Is, Zachary Quicksall, Merve Atik, Joseph S. Reddy, Venkata N.S. Duvuuri, Yuhao Min, Junli Gao, Katie D Sotelo, Thuy Nguyen, Dennis W. Dickson, Mariet Allen, Nilüfer Ertekin‐Taner

**Affiliations:** ^1^ Mayo Clinic, Jacksonville, FL USA; ^2^ Mayo Clinic Graduate School of Biomedical Sciences, Rochester, MN USA

## Abstract

**Background:**

A complex, multicellular disease with genetic and immunological elements, Alzheimer’s disease (AD) affects millions worldwide. There has been previous research linking AD to the missense variants ABI3‐rs616338‐T and PLCG2‐rs72824905‐G, and the altered expression of these genes has been shown to disrupt microglial function. In our understanding of AD risk and resilience, limited research has been conducted on how these variants affect microglial subtypes and states in AD.

**Methods:**

We previously identified DOCK8 protein as a target for fluorescent activated nuclei sorting (FANS) to enrich microglia nuclei from frozen human brains. Using this enrichment strategy, we generated microglia enriched snRNAseq data from temporal cortex tissue of 30 donors harboring *ABI3* or *PLCG2* missense mutations, or neither mutation. We introduced *PLCG2* variant in either homozygote, and heterozygote forms into an AD patient derived iPSC through CRISPR/Cas9 approach, differentiated these cells into microglia cells (IMGLs) following established protocols, and generated scRNAseq data after treatment with Amyloidβ. We applied standard alignment and quality control pipelines to the snRNAseq and scRNAseq datasets in parallel. Established markers of microglia states and subtypes were utilized to annotate the clusters; analyzed for their hub gene expression using comparisons between cluster differential profiling to determine signature pathways. Differential gene expression was assessed using pseudo bulk and MAST approaches.

**Results:**

We obtained single nuclei transcriptome profiles of 54,000 frozen human brain nuclei, of which 35,000 are microglia, through snRNAseq, and single cell profiles of 63,000 iMGLs through scRNAseq, using our standard alignment and quality control procedures. Our differential gene expression analysis identified novel genes and pathways which were identified as part of resilience or AD risk networks in microglia subtypes and states. These alterations were validated using orthogonal experimental methods and further explored for their conservation using *in vivo* external datasets. Pseudotime inference will be completed to probe microglia marker gene expression dynamics, treatment conditions, along with continuous cell‐state changes.

**Conclusion:**

Our study uncovers microglia subtype specific resilience and risk factors provided by AD related genetic variants using snRNAseq from frozen human brain and scRNAseq from IPSC derived microglia cells. These findings nominate novel targets and pathways with therapeutic potential.

